# Effect of desertification on productivity in a desert steppe

**DOI:** 10.1038/srep27839

**Published:** 2016-06-14

**Authors:** Zhuangsheng Tang, Hui An, Lei Deng, Yingying Wang, Guangyu Zhu, Zhouping Shangguan

**Affiliations:** 1 State Key Laboratory of Soil Erosion and Dryland Farming on the Loess Plateau, Northwest A&F University, Yangling 712100, P.R. China; 2 Key Laboratory of Restoration and Reconstruction of Degraded Ecosystems in North-western China of the Ministry of Education, United Center for Ecology Research and Bioresource Exploitation in Western China, Ningxia University, Yinchuan 750021, P.R. China

## Abstract

Desertification, one of the most severe types of land degradation in the world, is of great importance because it is occurring, to some degree, on approximately 40% of the global land area and is affecting more than 1 billion people. In this study, we used a space-for-time method to quantify the impact of five different desertification regimes (potential (PD), light (LD), moderate (MD), severe (SD), and very severe (VSD)) on a desert steppe ecosystem in northern China to examine the relationship between the productivity of the vegetation and soil properties and to determine the mechanism underlying the effects of desertification on productivity. Our results showed that the effects of desertification on TP (total phosphorus) and AP (available phosphorus) were not significant, and desertification decreased productivity in the desert steppe as a result of direct changes to soil physical properties, which can directly affect soil chemical properties. Therefore, intensive grassland management to improve soil quality may result in the long-term preservation of ecosystem functions and services.

Desertification, one of the most severe types of land degradation in the world, is of great importance because it is occurring, to some degree, on approximately 40% of the global land area and is affecting more than 1 billion people^1^. Stopping or reversing this process is crucial because desertification not only results in soil degradation and decreased soil productivity^2^, but it also changes ecosystem function and structure^3^.

Above-ground productivity, which correlates with key aspects of ecosystem function, has been used as an indicator of desertification^4^, but whether productivity is hampered by a lack of water remains a matter of debate^5^. Previous studies have reported that ANPP (the aboveground net primary production) is extremely heterogeneous, both temporally and spatially^6^, so the question of whether productivity decreases in conjunction with desertification remains a central issue in the study of the process^4^.

In arid and semiarid regions, wind erosion^7^ and over-grazing^8^ are the principal drivers of land deterioration^2^,^9^, and recent evidence suggests that wind erosion results in the heterogeneous distribution of soil nutrients^10^. Su *et al*.^11^ focused on the effects of grazing on soil properties and found that over-grazing changed soil texture and the distribution of soil particle size in a sand grassland. Pei *et al*.^12^ observed that over-grazing resulted in decreased productivity, which is valuable for identifying cause–effect relationships to improve our understanding of the changes in soil and vegetation characteristics during land degradation. Recent critical load experiments have focused on individual changes in the soil or vegetation as a consequence of desertification, but these experiments have limitations and are poorly suited for elucidating the mechanisms underlying changes in the productivity of desert steppe undergoing desertification. Understanding the ecological effects of desertification on arid ecosystems is essential, and an understanding of the relationship between grassland productivity and soil properties is necessary for the long-term sustainable management of grassland ecosystems.

To maintain the sustainability of desert steppe ecosystems, it is necessary to understand their vegetation degradation dynamics relative to their soil properties. In this study, we used a space-for-time method to study the relationship between vegetation productivity and soil properties and the mechanism underlying the effects of desertification on productivity in the desert steppe ecosystem of Ningxia, China. The objective was to estimate the effects of desertification on soil properties and to analyze the factors affecting vegetation productivity to explain the mechanism underlying variations in the vegetation of the desert steppe ecosystem of northern China.

## Results

### Changes to above- and below-ground and litter biomass

**Figure 1:**
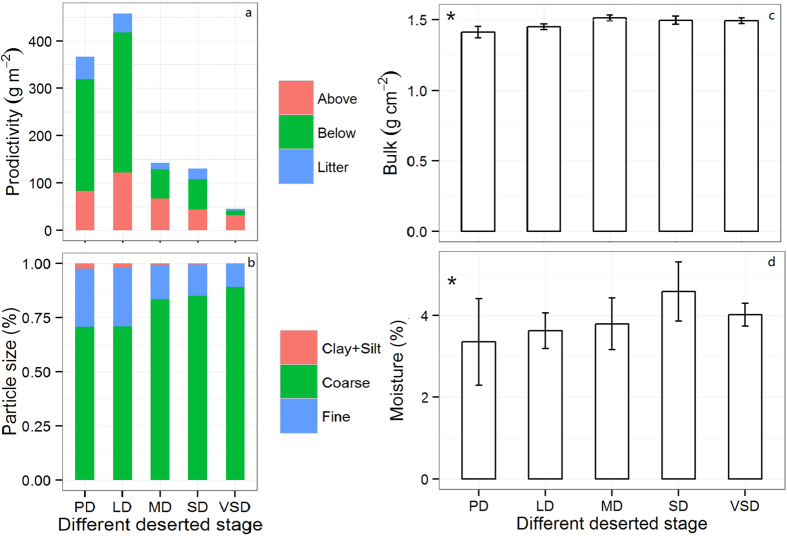
Changes in vegetation biomass and soil properties in different stages of desertification. (**a**) Above ground biomass, litter biomass, and below ground biomass; (**b**) soil mechanical composition; (**c**) soil bulk density; (**d**) soil moisture content. PD, potential desertification; LD, light desertification; MD, moderate desertification; SD, severe desertification; VSD, very severe desertification. The values are the mean ± SE. Significant differences between the different varieties are indicated by the symbol *p < 0.05.

An analysis of variance of 3 vegetation variables indicated that grassland desertification has a significant effect on vegetation productivity (P < 0.05). Above- and below-ground biomass and litter biomass decreased linearly as desertification intensified. But above- and below-ground biomass increased by 47% and 25%, respectively (Fig. 1a), when comparing the LD (light desertification) stages with the PD (potential desertification), and the highest above- and below-ground biomass values in the LD stage were 122.01 and 295.94 g m^−2^, respectively. The above- and below-ground biomass and the litter biomass decreased by 61.9%, 95.9%, and 92.1%, respectively, in the VSD stage compared with the PD stage (Fig. 1a), which indicates that grassland desertification has a greater effect on below-ground biomass than on above-ground and litter biomass.

### Changes in the physical properties of the soil

As seen in Fig. 1b, the grasslands experienced an intensifying degree of desertification; the coarse sand content increased significantly (*P* < 0.01). Compared with the PD stage, the coarse sand content in the VSD (very severe desertification) stage increased by 26%, but the clay-silt and fine sand content tended to decrease the most (*P* < 0.01).

Across the different grassland desertification stages, soil bulk density initially increased and then subsequently decreased. The maximum and minimum soil bulk density values were 1.51 g cm^−2^ and 1.41 g cm^−2^ in the MD (moderate desertification) and PD stages, respectively (Fig. 1c). As seen in Fig. 1d, the desert steppe ecosystem had low moisture content; the maximum value was 4.6%. Soil bulk density and soil moisture showed similar tendencies (Fig. 1c,d), but the maximum value appeared in the SD stage. Soil bulk density was more sensitive to desertification than soil moisture.

### Changes in the chemical properties of the soil

**Figure 2:**
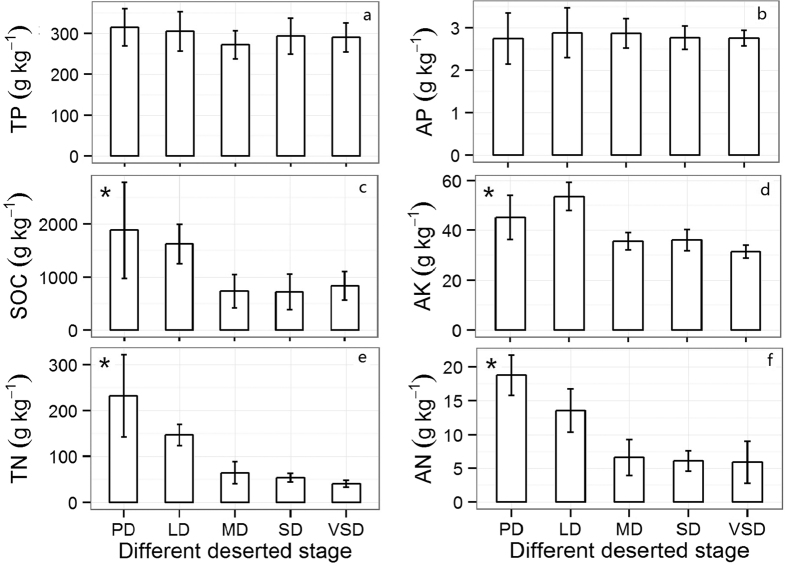
Changes in soil chemical properties in different stages of desertification. (**a**) TP (total phosphorus); (**b**) AP (available phosphate); (**c**) SOC (soil organic carbon); (**d**) AK (available potassium); (**e**) TN (total nitrogen); (**e**) AN (available nitrogen). PD, potential desertification; LD, light desertification; MD, moderate desertification; SD, severe desertification; VSD, very severe desertification. The values are the mean ± SE. Significant differences between different varieties are indicated by the symbol *p < 0.05.

The change in soil nutrient content was significant (*P* < 0.05) as desertification intensified, except for the concentrations of TP (total phosphorus) and AP (available phosphate) (*P* > 0.05). Both SOC (soil organic carbon) and AN (available nitrogen) rapidly decreased through the different stages of grassland desertification and tended to be stable after the MD stage. The highest SOC, TN (total nitrogen), and AN contents were found in the PD stage, except for the AK (available potassium) content in LD. The contents of SOC, AK (available potassium), AN and TN decreased in the VSD stage by 70.1% and 30.4%, 68.8% and 82.4%, respectively (Fig. 2), compared with the PD stage, indicating that desertification had a greater impact on the supply of soil nutrients.

### Community productivity in relation to soil properties

**Figure 3:**
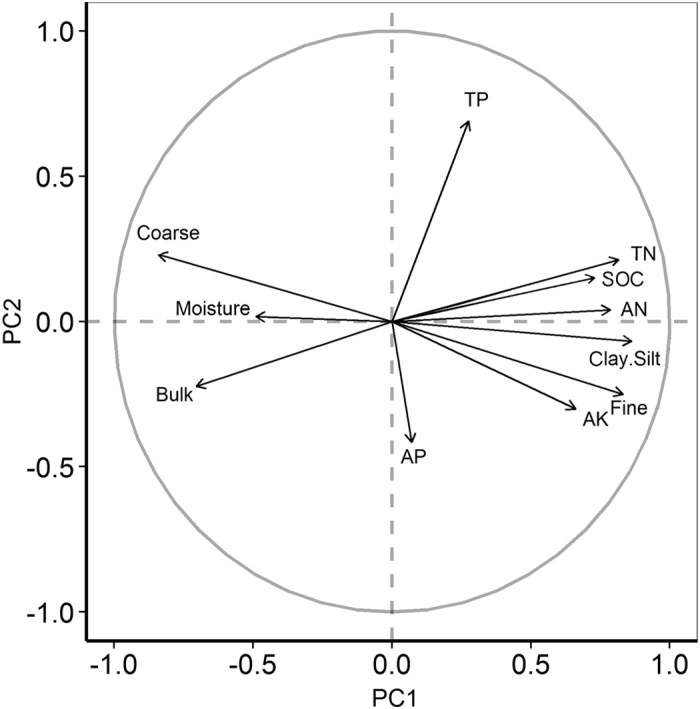
Principal components analysis of 11 soil variables; each arrow represents the eigenvector corresponding to an individual variable. PC1 accounted for 59.2% of the overall variance, and PC2, for 11.2% of the overall variance.

A principal components analysis (PCA) of 11 soil variables was used to represent the correlations among the variables of the first two principal axes associated with the first two principal components (Fig. 3). PCA axis 1 primarily reflected the chemical properties of the soil and accounted for 59.2% of the overall variance in the standardized soil variables; axis 2 mainly reflected the physical properties of the soil, explaining 11.2% of the standardized variance. As seen in Fig. 3, the clay-silt and fine sand content of the soil were negatively correlated with coarse soil content, so we used the inverse of the clay-silt and fine sand values to conduct the following analysis. A Monte Carlo permutation test was used to test the significance of all of the environmental factors, and the results showed that all of the soil variables were significant except for TP and AP, which were excluded in the following analysis.

### Structural equation model of the effects of soil properties on vegetation productivity

**Figure 4:**
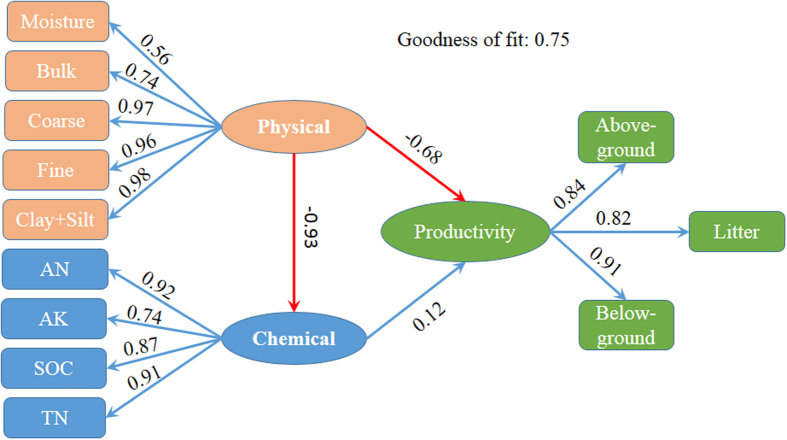
Structural equation model of productivity, soil physical properties, and chemical properties. The standardized coefficient is given for SEM. Values in rectangular frames denote the measurable variables. Values in ellipse frames denote the latent variables. Goodness of fit was 0.75 for SEM. Red arrows denote negative correlation. Blue arrows denote positive correlation. The inverse of the variable value of clay + silt and fine sand was used to conduct the SEM.

According to the results of the PCA, the physical (including moisture, soil bulk, coarse sand, fine sand, and clay + silt sand) and chemical properties (including AN, AK, SOC, and TN) of the soil and vegetation productivity (including above-ground, below-ground, and litter biomass) served as the three latent variables to establish a structural equation model. The structural equation model of the environmental and productivity factors showed that both the chemical and the physical properties of the soil affected the productivity of the grassland vegetation. The goodness of fit index was 74.52, indicating that this model explained the changes in vegetation productivity well. The physical and chemical properties accounted for 46.8% of the variance in productivity (Fig. 4).

However, the variation in the physical properties appeared to have a much stronger impact on desert steppe productivity than nutrient supply. The physical properties of the soil had a significant negative influence on productivity, and the total effect was −0.80 (P < 0.01), of which the direct effect was −0.68 and the indirect effect was −0.12. The effect of the soil chemical properties on productivity was not significant (*P* > 0.05, Fig. 4).

The soil physical properties also have a significant negative effect on the soil chemical properties; the direct effect was −0.93 (*P* < 0.01). The physical properties accounted for 64.4% of the variance in chemical properties (Fig. 4).

For our structural equation model, the loadings of all of the variables, except soil moisture, were above 0.7 (Fig. 4).

## Discussion

In this study, grassland desertification markedly reduced vegetation biomass by breaking apart large patches of vegetation by destructive measures (e.g., grazing and wind erosion). As vegetation biomass decreases and the soil becomes more exposed, the risk of desertification increases^7^,^13^. Interestingly, we found that desertification leads to decreases in vegetation biomass and that the dominant species in the LD stage, which has the greatest biomass, was *Sophora alopecuroides*, which can contribute to the symbiotic fixation of N. Therefore, it would be expected that this fixation would have a positive effect on biomass^14^.

Soil textural properties are mainly inherited from parent materials and are resistant to change^15^, but our data regarding the mechanical composition variables showed that fine soil particles (e.g., fine sand and clay + silt sand) decreased and that soil texture declined with intensifying desertification. Such patterns were consistent with the observations of Tang *et al*. and Zhou *et al*.^16^,^17^, but we did not find a similar pattern to that of previous research^18^. Soil water moisture content has an important effect on vegetation development^15^, and our results showed that soil moisture was not a major factor limiting productivity in the desert steppe, which was consistent with the study by Austin^5^. Many studies have also shown that desert steppe vegetation degradation may raise the groundwater table and increase the soil moisture content^15^,^19^ because evapotranspiration is lower in bare areas than in vegetated areas. As a result, bare areas are often moist beneath the surface. In addition, we found that changes in soil bulk density and soil moisture with grassland desertification were not consistent, with soil bulk density being more sensitive to desertification than soil moisture content. These results indicated that soil texture has a strong effect on soil moisture, and PCA showed that there was a significant correlation among soil moisture, soil bulk density, and clay + silt sand content.

Generally, soil nutrients appear to be variable in arid and semiarid area ecosystems because many variables interact with and contribute to the spatial distribution of soil nutrients^20^. Our results showed that soil nutrient content decreased with intensifying desertification, which suggests that desertification results in a more heterogeneous distribution of soil nutrients and that soil becomes barren as desertification progresses. However, we found that changes in TP and AP were not significant because drought conditions may decrease the mass flow or diffusivity of nutrients, thereby restricting nutrient transport and, as a result, P uptake^21^. The diffusivity of P in the soil is more sensitive to soil moisture than that of other nutrients (e.g., N)^22^.

Although a few studies have examined changes in productivity in arid and semiarid grasslands^4^,^12^,^23^, none have investigated the underlying mechanism in terms of the role of soil physical and chemical properties. It is natural to assume that compositional and structural variations in physical and chemical properties would be synchronous, but the following question remains: Do soil physical properties play a greater role than nutrient supply in determining plant community productivity in desert steppe ecosystems? SEM provided evidence that soil physical properties, which are clearly correlated with soil chemical properties, were the critical influencing factor leading to a decrease in grassland productivity following desertification. Changes in the soil physical properties were the main reason for the variance in soil chemical properties. Spatial heterogeneity of soil properties is a common feature in natural ecosystems^19^,^24^, and the initiation of such heterogeneity has primarily been explained by wind erosion and over-grazing, which alter the relatively uniform distribution of soil moisture, bulk density, and soil particle composition^7^,^10^,^12^. On the other hand, climate change is the driving force for land degradation and carbon emissions from soils^4^, and PCA showed that changes in SOC and TN content were significantly positively correlated with soil fine particle content and negatively correlated with coarse sand content and bulk density. A previous study by Zhao *et al*.^25^ also found that fine soil particles contain more SOC and TN. In addition, SEM showed that physical properties accounted for 64.4% of the variance in chemical properties, and the path coefficient was −0.93 (*P* < 0.05). These results indicated that the nutrient-rich fine soil particles decreased due to soil texture degradation. We found that the physical and chemical properties accounted for 46.8% of the variance in productivity. However, the effect of chemical properties on productivity was non-significant (*P* > 0.05), and the physical properties had a negative effect on productivity. Thus, we concluded that desertification decreased productivity in the desert steppe as a result of direct changes in the soil physical properties, which can have direct effects on soil chemical properties. Therefore, intensive grassland management to improve soil quality may provide an effective way to ensure the long-term preservation of ecosystem functions and services. However, ecosystems are subject to a dynamic balancing process, so productivity is also influenced by temperature, precipitation, clouds and atmospheric aerosols, especially in arid and semi-arid areas^4^,^26^,^27^,^28^. However, this study did not involve these factors, so the pattern of change in productivity should be further explored in future research.

## Methods

### Study area

**Figure 5:**
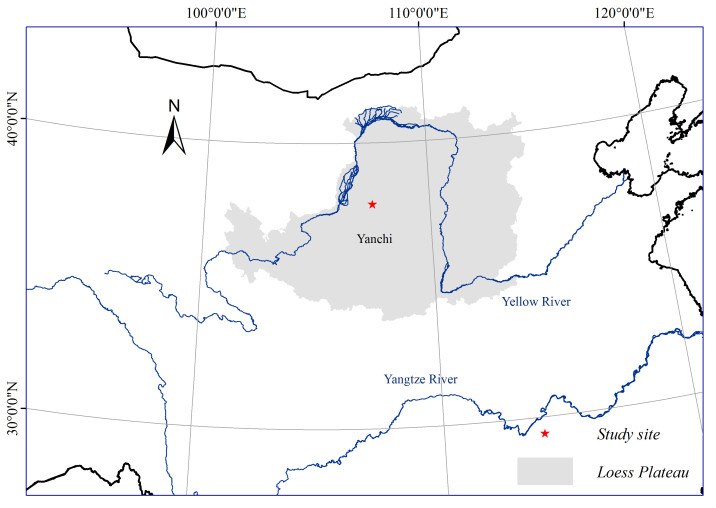
Location of the study area. The pictures were generated by ArcMap Version 10.2 (http://www.esri.com/).

**Figure 6:**
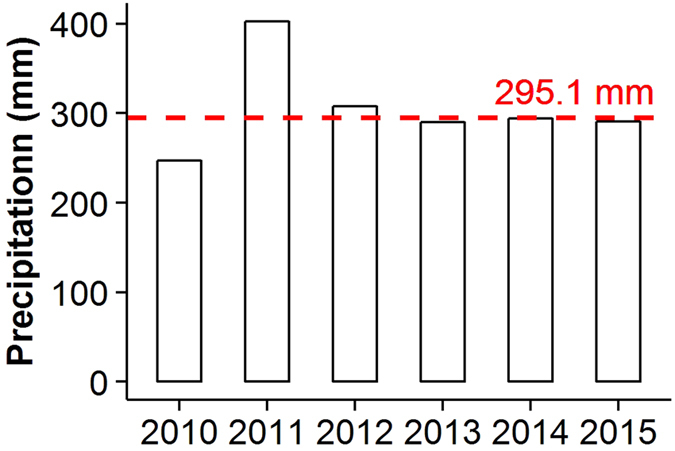
Precipitation at the study site from 2010 to 2015. Red dashed line denotes average precipitation from 1954 to 2014.

The study was conducted in Yanchi County (37 04′–38 10′N and 106°30′–107°41′E at an elevation of 1450 m, Fig. 5) on the southwestern fringe of the Mu Us sandy land in Ningxia, China, in the central and western regions of the Loess Plateau. The climate is semi-arid temperate with a mean annual temperature of 8.1 °C and minimum and maximum mean monthly temperatures of −8.7 °C in January and 22.4 °C in July. The average precipitation is 295.1 mm (Fig. 6), with 70% of the total precipitation occurring between June and September; Figure 6 shows the annual precipitation from 2010 to 2015. The average potential pan evaporation is 2014 mm per year. The average annual wind velocity is 2.8 m s^−1^, and the prevailing winds are mainly northwesterly in April and May. Sand particles are blown at velocities greater than 5.0 m s^−1^, on average, 323 times per year, and wind erosion often occurs from April to mid-June before the beginning of the rainy season (climate data from Yanchi Meteorological Station, 1976–2010).

At the study site, the main soil types are sierozem, loess and orthi-sandic entisols, all of which have low fertility and loose structure and are very susceptible to wind erosion^29^. The predominant vegetation in the mobile sand land is *Agriophyllum squarrosum*, but as the mobile sand land is gradually stabilized, the herbaceous vegetation is dominated by *Corispermum hyssopifolium*, *Salsola collina*, *Artemisia scoparia*, *Aneurolepidium dasystachys*, *Pennisetum centrasiaticum*, and *Cleistogenes gracilis*.

### Sampling and measurements

#### Experimental design

We used a space-for-time method in this study. According to the vegetation cover, study sites were chosen that were far from fencing or roads and showed a lower degree of anthropogenic interference. Five sampling areas across four different desertification stages were randomly chosen along with one area devoid of desertification (Ding, 2004). The stages were potential desertification (PD), light desertification (LD), moderate desertification (MD), severe desertification (SD), and very severe desertification (VSD); the PD stage served as the control. We selected 5 study sites for each stage of desertification, each of which exceeded 100 × 100 m (and were located approximately 200 m away from each other). At each site, ten 1 × 1-m quadrats were randomly established.

#### Biomass measurement

A field survey was undertaken each year from 2010 to 2015 between July and August, when biomass had reached its peak. In each quadrat, the above- and below-ground and litter biomass were investigated. The above-ground parts of the green plants were cut, sorted into envelopes by species and then tagged, and all of the litter was also collected, placed into envelopes and tagged. All of the above-ground parts of the green plants were immediately dried for 30 minutes at 105 °C and then transferred to the laboratory, where they were oven-dried at 65 °C and weighed.

After collecting the above-ground parts of the green plants and the litter, a 9-cm diameter root augur was used to collect three soil samples from each of the indicated depths (0–10, 10–20, 20–30, 30–40, 40–60, 60–80, and 80–100 cm) in each quadrat to measure the below-ground biomass. Samples taken from the same layer were then mixed to create a composite sample. The majority of the roots were found in these soil samples and isolated using a 2-mm sieve, and the remaining fine roots were removed from the soil samples and isolated by spreading the samples in shallow trays. Each tray was overfilled with water, and the outflow was allowed to pass through a 0.5-mm mesh sieve. No attempts were made to distinguish between living and dead roots. All of the roots were immediately dried for 30 minutes at 105 °C and then transferred to the laboratory, where they were oven-dried at 65 °C and weighed. The data for the total amount of below-ground biomass in the 0–100 cm layers were analyzed in this paper.

#### Soil sampling

Soil samples for chemical analysis were collected at three points in each quadrat: the two corners and the center along the diagonal from which root samples were not collected. The soil samples were passed through a 2-mm screen to remove roots and other debris, and each sample was air-dried and stored at room temperature until its physical and chemical properties could be determined. Using a soil bulk sampler with a 5-cm diameter and 5-cm high stainless steel cutting ring, the soil bulk density of each soil layer (0–10, 10–20, 20–30, 30–40, 40–60, 60–80, and 80–100 cm) was also measured (three replicates) at points adjacent to where the soil samples for the chemical analysis were collected. The original volume of each soil core and its dry mass after oven-drying at 105 °C were measured.

The data for the average soil moisture in the 0–100 cm layers were analyzed in this paper.

#### Chemical determination

Soil samples from the 0–20-cm layer were used for chemical determination; replicate soil cores from each sampling site were mixed and sieved (through a 2 mm mesh screen). The SOC (soil organic carbon) content was assayed via dichromate oxidation^30^, and the soil TN (total nitrogen) content was assayed using the Kjeldahl method^31^. Soil AN (available nitrogen) content was extracted using hot H_2_O_2_/KCl^32^. Soil TP (total phosphorus) content was determined after digestion with HClO^−^–H_2_SO_4_^33^, and the AP (available phosphate) was determined by the molybdenum blue method^34^. Concentrations of AK (available potassium) in the soil were determined using atomic absorption and flame emission spectrometry after extracting the soil with NH_4_OAc solution^35^. Each of the analyses was performed in duplicate.

### Statistical analysis

The data from 2014 were analyzed in this study. All data were analyzed using R version 3.1.0 (https://www.R-project.org/), and an ANOVA (one-way analysis of variance) was performed to identify significant differences among the treatments. Given the strong correlations between several soil parameters, we used the “plsdepot” package^36^ to conduct a principal components analysis (PCA) of standardized values of those parameters to identify the primary axes of covariation among them. Then, we used a Monte Carlo permutation test of the significance of the PCA effects, and we eliminated the variables TP and AP, which were not significant. The partial least squares approach was used to conduct SEM (structural equation modeling) with productivity, the soil physical properties, and the soil chemical properties as the latent variables. SEM was conducted with the “plspm” package in R Version 3.1.0^36^. Significant differences at *P* < 0.05 were evaluated at a 95% confidence level.

## Additional Information

**How to cite this article**: Tang, Z. *et al*. Effect of desertification on productivity in a desert steppe. *Sci. Rep.* **6**, 27839; doi: 10.1038/srep27839 (2016).
